# The efficacy and safety of intralesional injection of collagenase *Clostridium histolyticum* for Peyronie’s disease: A meta-analysis of published prospective studies

**DOI:** 10.3389/fphar.2022.973394

**Published:** 2022-10-05

**Authors:** Fuxun Zhang, Yang Xiong, Wei Wang, Changjing Wu, Feng Qin, Jiuhong Yuan

**Affiliations:** ^1^ Andrology Laboratory, West China Hospital, Sichuan University, Chengdu, Sichuan, China; ^2^ Department of Urology, West China Hospital, Sichuan University, Chengdu, Sichuan, China

**Keywords:** Peyronie’s disease, collagenase *Clostridium histolyticum*, treatment, efficacy, safety

## Abstract

**Background:** Peyronie’s disease (PD) is a progressive fibrotic disorder of the penis that is adverse to men’s health. Currently, effective and reliable non-surgical options for PD are limited. Since the Food and Drug Administration (FDA) approved it in 2013, intralesional injection of collagenase *Clostridium histolyticum* (CCH) became the only licensed treatment for PD. This meta-analysis aims to evaluate the clinical efficacy and safety of CCH in treating PD, predominantly based on post-FDA studies.

**Methods:** The primary outcome was clinical efficacy evaluated by the percentages of improvement in penile curvature (PC) and Peyronie’s disease symptom bother score (PD bother score). The secondary outcome was the safety assessed by treatment-related adverse events (TRAEs). Heterogeneity was assessed by Cochran’s Q and *I* ^*2*^ tests. Sensitivity and subgroup analyses were performed to explore the source of heterogeneity. Funnel plots and Egger’s test were used to evaluate the publication bias.

**Results:** A total of 11 studies with 1,480 intentions to treat (ITT) population were included. The pooled effect of the improvement of PC was 35% (95% CI: 0.33–0.38), and the pooled improvement of the PD bother score was 41% (95% CI: 0.37–0.45). No heterogeneity was found at the pooled improvement of PC (*p* = 0.845, *I* ^*2*^ = 0.00%). Meanwhile, some heterogeneity existed in the pooled improvement of the PD bother score (*p* = 0.069, *I* ^*2*^ = 43.4%). The pooled effect of TRAEs was 93% (95% CI 0.88–0.97) with significant heterogeneity (*p* < 0.000, *I* ^*2*^ = 92.3%).

**Conclusion:** The intralesional injection of CCH could significantly improve the penile deformity of PD patients. Meanwhile, CCH appears to ameliorate the PD bother score to some extent and has acceptable clinical safety. Future studies are required to clarify the long-term outcomes of CCH injection in the treatment of PD.

## Introduction

Peyronie’s disease (PD) is a progressive penile disorder characterized by fibrotic plaque or scar formation in the tunica albuginea (Russo et al., 2018). PD can provoke local pain, penile curvature deformity, and intercourse difficulty, which have an adverse effect on psychological and sexual function of patients. The prevalence of PD is reportedly from 22.4 to 25.7 per 100,000 men, and the average age of patients is 55 years (Herati and Pastuszak, 2016). Actually, the incidence of PD might be underestimated due to under-reporting bias from those patients who are reluctant to visit hospitals (Dhillon, 2015). As of yet, surgery is considered the gold-standard treatment of PD in the stable phase to provide patients with permanent functional recovery and correction of penile deformity (Wymer et al., 2019). However, including intralesional injection and mechanical treatment, there are several non-surgical options are aimed at slowing down the fibrotic process, decreasing curvature deformity, and improving erectile function before surgery (Tsambarlis and Levine, 2019).

Collagenase *Clostridium histolyticum* (CCH), a mixture of class-I and class-II clostridial collagenases (AUX-I and AUX-II) possessing similar and complementary substrate specificity, has been proven to degrade the essential fibrotic composition in PD plaques and collagen types I and III (Dhillon, 2015; Anaissie et al., 2016). Thus, in 2010, Investigation for Maximal Peyronie’s Reduction Efficacy and Safety Studies (IMPRESS) I and II, two large randomized controlled trials (RCTs) in phase III were performed and subsequently demonstrated significant improvement in curvature deformity and PD bother domain score of the PD questionnaire (PDQ) (Chung et al., 2016). Furthermore, the Food and Drug Administration (FDA) approved the intralesional injection of CCH for the management of adult PD patients with palpable plaques and penile curvature ≥30° in 2013 (Peak et al., 2015). However, very few post-FDA studies conducted a comprehensive evaluation on the efficacy and safety of intralesional CCH for the treatment of PD. Meanwhile, a better understanding of the management of PD with standardized or shortened intralesional protocol is still required.

In this setting, we conducted a meta-analysis based on available prospective studies from IMPRESS I/II to now. This study aims to evaluate the clinical efficacy and safety of intralesional therapy of CCH on PD patients.

## Methods

### Search strategy

This meta-analysis was conducted in line with the PRISMA guidelines, and the PRISMA 2009 checklist was used (Liberati et al., 2009). The included studies were selected by searching PubMed, Embase, and Cochrane Library databases published from 2010 to 2021. The search terms were “Peyronie’s disease [MeSH Terms]” OR “Peyronie’s disease” OR “fibrous cavernitis” OR “plastic induration of the penis” OR “fibromatosis, penile” OR “penile fibromatosis” AND “Collagenase Clostridium Histolyticum [MeSH Terms]” OR “Nucleolysin” OR “Collagenase, Microbial” OR “Clostridiopeptidase” OR “Collagenase-Like Peptidase” OR “Collagenase-Like Peptidase.” The searches were performed within the limits of the English language, prospective design, and clinical trials.

### Inclusion criteria and data extraction

The inclusion criteria in this meta-analysis were as follows: (a) all studies are clinical trials with the prospective design; (b) the language of publication is English; (c) studies exclusively enrolled patients with PD whose age ≥18 years; (d) studies enrolled patients in the stable phase and with lateral or dorsal penile curvature between 30° and 90°; and (e) information about clinical efficacy or safety was available. Meanwhile, principal exclusion criteria included: (a) enrolled patients with extensive plaque calcification, pure ventral penile curvature, serious hourglass deformity, compromised penile hemodynamics, and other contraindications for CCH; (b) treatment protocol is not IMPRESS or modified shortened protocol; and (c) patients with receipts of previous surgery or intralesional therapies within 3 months of the study for PD. Two authors (YX and CJW) independently reviewed and extracted the data from the included studies, and the third author (FQ) was designated to resolve any discrepancies in this section.

### Outcomes and statistical analysis

The primary endpoint was clinical efficacy evaluated by the percentages of improvement in penile curvature (PC), and the percentages of improvement in the Peyronie’s disease symptom bother score (PD bother score) of the PD questionnaire (PDQ) validated by the FDA. The secondary outcome was the safety assessed by the incidence of treatment-related adverse events (TRAEs). Improvements or TRAEs of PD were considered the numerator and baseline characteristics as the denominator for calculating the percentages. Freeman−Tukey arcsine transformations were conducted for the rate equal to 1 or 100%. The included studies were reviewed by co-authors to validate all entries.

The pooled percentages with 95% confidence intervals (CIs) of each endpoint were selected as the evaluation indicators. The fixed- or random-effects model was used to account for heterogeneity. The effects of the outcome were first pooled with a fixed-effects model, and if heterogeneity was significant, the meta-analysis was also renewed with the random-effects model. Heterogeneity among included studies was assessed by Cochran’s Q and *I* ^*2*^ tests. Results with *I* ^*2*^ greater than 50% were considered to be heterogeneous. Meanwhile, sensitivity and subgroup analyses were performed to explore the major source of heterogeneity. In subgroup analysis, included studies were stratified by variables related to clinical factors, including treatment protocols and baseline characteristics. Funnel plots and Egger’s test were used to evaluate the publication bias and funnel plot asymmetry, respectively. Additionally, if a primary endpoint of included study was missing or inapplicable for synthesizing, it would be excluded in the corresponding subsection analysis. STATA software (version 15.1, Stata Corp, College Station, TX, USA) was used to conduct this meta-analysis.

## Results

### Literature selection and characteristics of the included studies

**FIGURE 1:**
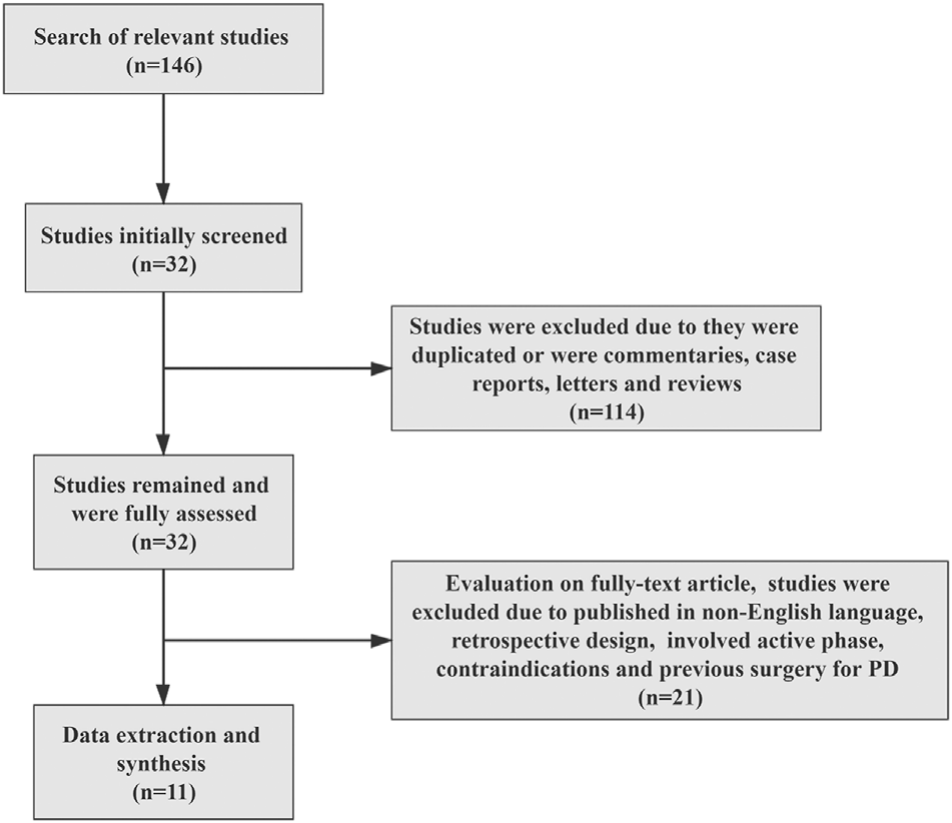
Flow chart of the included studies.

The process of literature selection is shown in Figure 1. Based on this, 146 studies were found through initial database searching. All in all, 114 studies were excluded according to the inclusion criteria, and the remaining 32 studies were fully assessed. Finally, a total of 11 studies with 1,480 intentions to treat (ITT) the population were included for data synthesis and meta-analysis (Table 1). The characteristics of the included studies are shown in Table 1. Among them, two studies conducted multi-institutional RCTs, and others adopted prospective designs. Meanwhile, two studies employed the modified shortened protocol for treatment.

**TABLE 1:** Characteristics of included studies.

Studies	Year	Title	Design	ITT	mITT	Age (years)	PD duration (years)
Gelbard M et al.	2012	Phase 2b study of the clinical efficacy and safety of collagenase Clostridium histolyticum in patients with Peyronie disease	Multi-institutional RCT	54	54	57.4 (36–72)	3.0 ± 2.8
Gelbard M et al.	2013	Clinical efficacy, safety and tolerability of collagenase clostridium histolyticum for the treatment of peyronie disease in 2 large double-blind, randomized, placebo controlled phase 3 studies	Multi-institutional RCT	551	401	59.0 (23–84)	4.1 ± 4.1
Levine LA et al.	2015	Clinical safety and effectiveness of collagenase clostridium histolyticum injection in patients with Peyronie's disease: a phase 3 open-label study	Prospective, open label, multi-center study	347	238	57.0 (23–77)	2.97 ± 2.8
Ziegelmann MJ et al.	2016	Restoration of Penile Function and Patient Satisfaction with Intralesional Collagenase Clostridium Histolyticum Injection for Peyronie's Disease	Prospective, non-randomized study	69	27	58.3 ± 9.1	2.1 ± 2.4
Raheem AA et al.	2017	Safety and effectiveness of collagenase clostridium histolyticum in the treatment of Peyronie's disease using a new modified shortened protocol	Prospective, single-centre study	53	53	54.0 (35–72)	1.9 ± 2.0
Goldstein I et al.	2017	Changes in the Effects of Peyronie’s Disease After Treatment With Collagenase Clostridium histolyticum: Male Patients and Their Female Partners	Prospective, open label, multi-center study	189	126	60.0 (33–77)	5.68 ± 3.2
Ralph DJ et al.	2017	Treatment of Peyronie's Disease With Collagenase Clostridium histolyticum and Vacuum Therapy: A Randomized, Open-Label Pilot Study	Prospective, randomized, open-label, pilot study	15	15	57.8 ± 9.4	NR
Ralph DJ et al.	2018	Treatment of Peyronie's Disease With Collagenase Clostridium histolyticum and Vacuum Therapy: A Randomized, Open-Label Pilot Study	Prospective, randomized, open-label, pilot study	15	15	57.6 ± 8.4	NR
Capece M et al.	2018	Collagenase clostridium histolyticum for the treatment of Peyronie's disease: a prospective Italian multicentric study	Prospective, non-randomized multi-centre study	135	135	54.4 (23–74)	1.1 (0.1–3.0)
Yafi FA et al.	2018	Multi-institutional Prospective Analysis of Intralesional Injection of ollagenase Clostridium Histolyticum, Tunical Plication, and Partial Plaque Excision and Grafting for the Management of Peyronie's Disease	Prospective, non-randomized multi-centre study	18	18	59.0 ± 6.2	1.0 (0.3–10.0)
Jensen CFS et al.	2020	Patient-reported outcomes from a single-centre prospective post-marketing study on Collagenase Clostridium Histolyticum injections for Peyronie's disease	Prospective single-centre study	34	27	62.0 (38–74)	1.17 (0.3–9.0)

Studies	Year	Treatment protocol	Concurrent treatment	Primary time of assessment	Mean baseline PC (°)	Baseline PD bother score	Improvement on PC (°)	Percentage of improved PC (%)	Improvement on PD bother score	Percentage of improved PD bother score (%)	TRAEs (%)	Serious TRAEs (n)
Gelbard M et al.	2012	IMPRESS protocol	Penile modeling/stretching	week 36	54.4 ± 15.1	8.6	17.5	32.4	3.6	41.9	96.4	0
Gelbard M et al.	2013	IMPRESS protocol	Penile modeling	week 52	50.1 ± 14.4	7.4	17.0	34.0	2.8	38.1	84.2	6
Levine LA et al.	2015	IMPRESS protocol	Penile modeling	week 24/36	53.0 ± 14.8	7.3	18.3	34.4	3.3	45.2	85.3	3
Ziegelmann MJ et al.	2016	IMPRESS protocol	Penile modeling/penile traction	week 24	63.0 ± 23.9	NR	22.6	37.8	NR	NR	86	0
Raheem AA et al.	2017	Modified shortened protocol	Penile modelling/stretching, vacuum therapy	week 12	54.2 ± 15.4	8.9	17.4	31.4	2.8	31.5	100	0
Goldstein I et al.	2017	IMPRESS protocol	Penile modeling	week 36	46.9 ± 12	6.3	17.0	36.3	2.4	38.1	93.1	3
Ralph DJet al.	2017	IMPRESS protocol	Penile modelling/stretching,vacuum therapy	week 36	59.0 ± 15.0	6.3	23.7	39.3	2.4	38.1	100	2
Ralph DJ et al.	2018	IMPRESS protocol	Vacuum therapy	week 36	58.3 ± 12.2	7.8	23.3	41.0	3.8	48.7	100	0
Capece M et al.	2018	Modified shortened protocol	Penile modelling/stretching, vacuum therapy	week 12	48.9 ± 22.3	11.9	19.1	42.9	4.44	37.2	92.6	0
Yafi FA et al.	2018	IMPRESS protocol	Penile modeling	week 24	65.5 ± 12.0	9.9	23.3	34.4	7.0	70.7	NR	0
Jensen CFS et al.	2020	IMPRESS protocol	Penile modeling/stretching	week 24	56.0 ± 20.0	11.1	NR	NR	4.6	41.4	NR	0

PD, Peyronie’s disease; PC, penile curvature; IMPRESS, Investigation for Maximal Peyronie’s Reduction Efficacy and Safety Studies; TRAEs, treatment-related adverse effects; ITT, intention to treat; mITT, modified intention to treat; RCT, randomized control trial; NR, not reported.

### Assessment of efficacy

**FIGURE 2:**
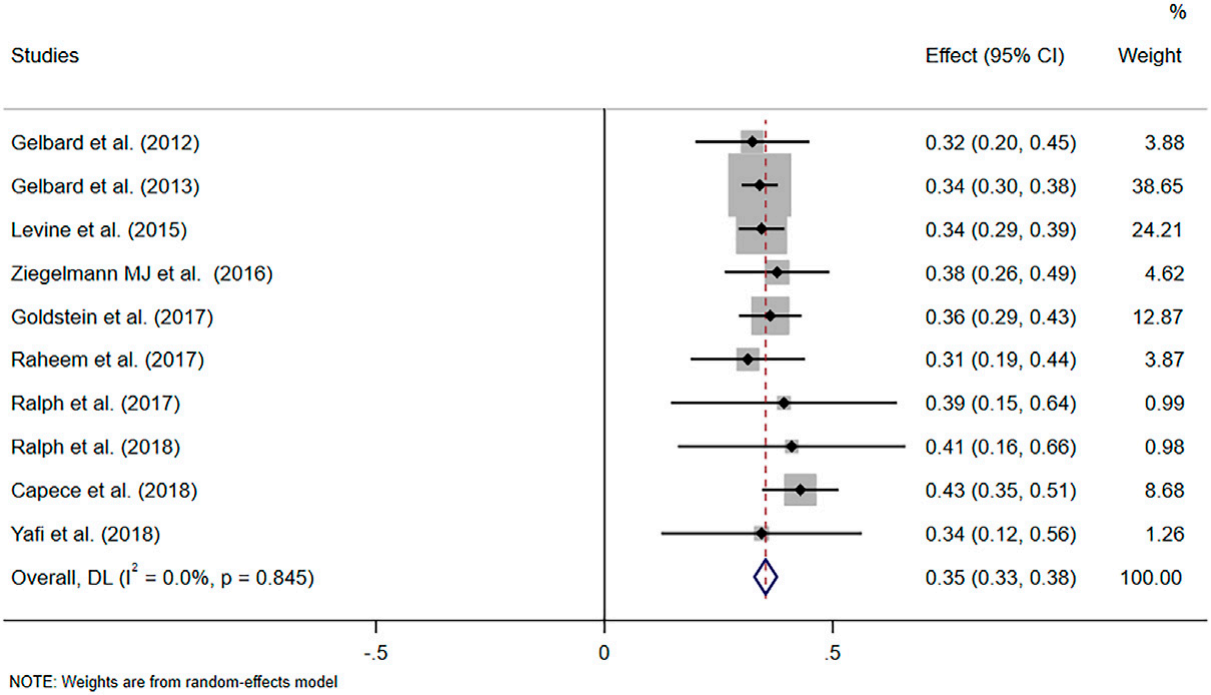
Forest plot of the pooled percentage of improved PC.

**FIGURE 3:**
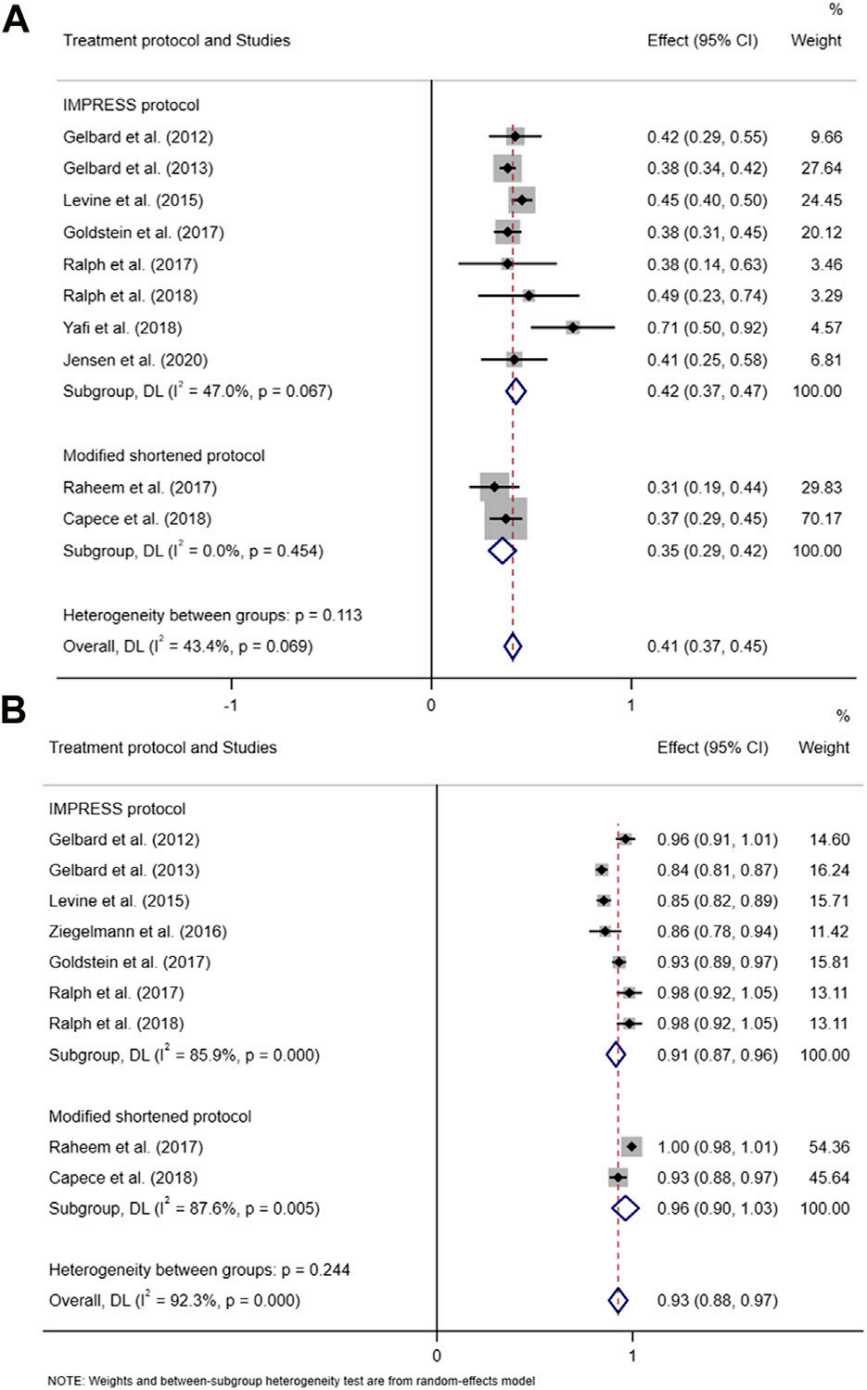
Forest plot of the pooled percentage of improved PD bother score based on subgroup analysis (stratified by the treatment protocol) **(A)** and the forest plot of the pooled TRAEs based on subgroup analysis (stratified by the treatment protocol) **(B)**.

The pooled data of the percentage of improved PC and PD bother score were obtained to evaluate the efficacy of CCH. Pooled effects of improvement in PC and PD bother score were 35% (95% CI 0.33–0.38) and 41% (95% CI: 0.37–0.45), respectively (Figure 2 and Figure 3A). Meanwhile, no heterogeneity was found at the pooled improvement of PC using fixed- and random-effects models (*p* = 0.845, *I* ^*2*^ = 0.00%). Furthermore, some heterogeneity existed in the pooled improvement of the PD bother score (*p* = 0.069, *I* ^*2*^ = 43.4%). The efficacy of CCH on the improvement of the PD bother score was assessed again for subgroups with different treatment protocols to determine a heterogeneity source. A random-effects model was used in subgroup analyses, and no heterogeneity between subgroups was shown (*p* = 0.113). Moreover, the pooled improvement of the PD bother score was 42% (95% CI 0.37–0.47) in the subgroup with IMPRESS protocols and 35% (95% CI 0.29–0.42) with the modified protocol. Among them, the subgroup with IMPRESS protocols presented more heterogeneity (*p* = 0.067, *I* ^*2*^ = 47%), whilst the subgroup with the modified shortened protocol presented little heterogeneity (*p* = 0.454, *I* ^*2*^ = 0.0%). We also assessed the improvement of the PD bother score based on the subgroup stratified by the median of baseline penile curvature (55°) with a random-effects model. Heterogeneity in the subgroup with a baseline curvature less than 55° (*p* = 0.209, *I* ^*2*^ = 30.2%) and heterogeneity in the subgroup with a baseline curvature more than 55° (*p* = 0.130, *I* ^*2*^ = 46.9%) were acceptable (Supplementary Figure S1). Additionally, we analyzed the other two domains of PDQ, namely, psychological and physical symptom score (PS score) and penile pain score (PP score), based on the studies that reported these two domains (Supplementary Figure S2). Despite the significant heterogeneity, it seems that intralesional therapy of CCH may be useful in improving the psychological and penile pain symptoms.

### Assessment of safety

Available data on TRAEs from nine included studies were pooled to evaluate the safety of CCH. The pooled effect of the percentage of TRAEs using random effect meta-analyses was 93% (95% CI 0.88–0.97). Significant heterogeneity was displayed in the pooled TRAEs (*p* < 0.000, *I* ^*2*^ = 92.3%) (Figure 3B). Thus, sensitivity analyses and subgroup analyses were performed to detect the potential reason for heterogeneity. However, heterogeneous distribution in the evaluation of safety is unsystematic and irregular (Supplementary Figure S3). Heterogeneity of the subgroup with the IMPRESS protocol or modified protocol was not significantly decreased (*p* < 0.000, *I* ^*2*^ = 85.9% and *p* = 0.005, *I* ^*2*^ = 87.6%, respectively). No consistency was found for all safety outcomes. Moreover, the risk of publication bias was high. Thus, the pooled estimation of safety might be influenced by some heterogeneous factors existing in the included studies.

### Publication bias

**FIGURE 4:**
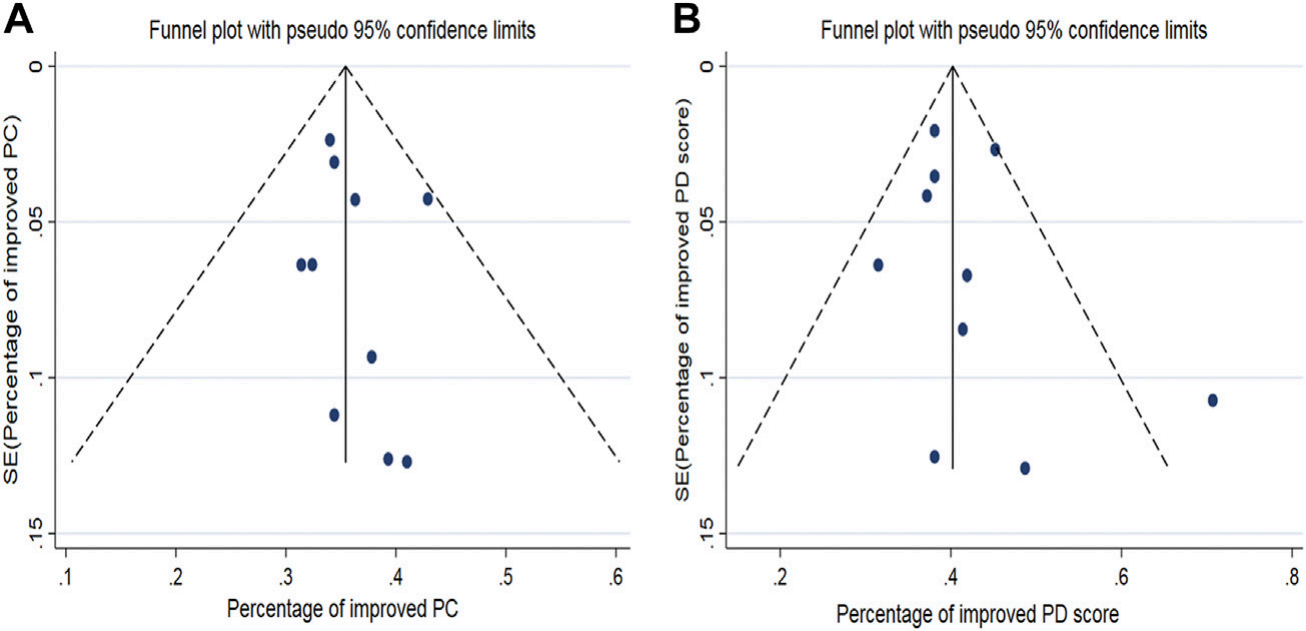
Funnel plot generated for publication bias of the pooled percentage of improved PC **(A)** and the pooled percentage of improved PD bother score **(B)**.

**FIGURE 5:**
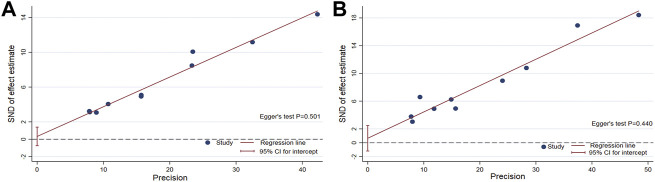
Egger’s test to measure the funnel plot asymmetry of the pooled percentage of improved PC **(A)** and the pooled percentage of improved PD bother score **(B)**.

**FIGURE 6:**
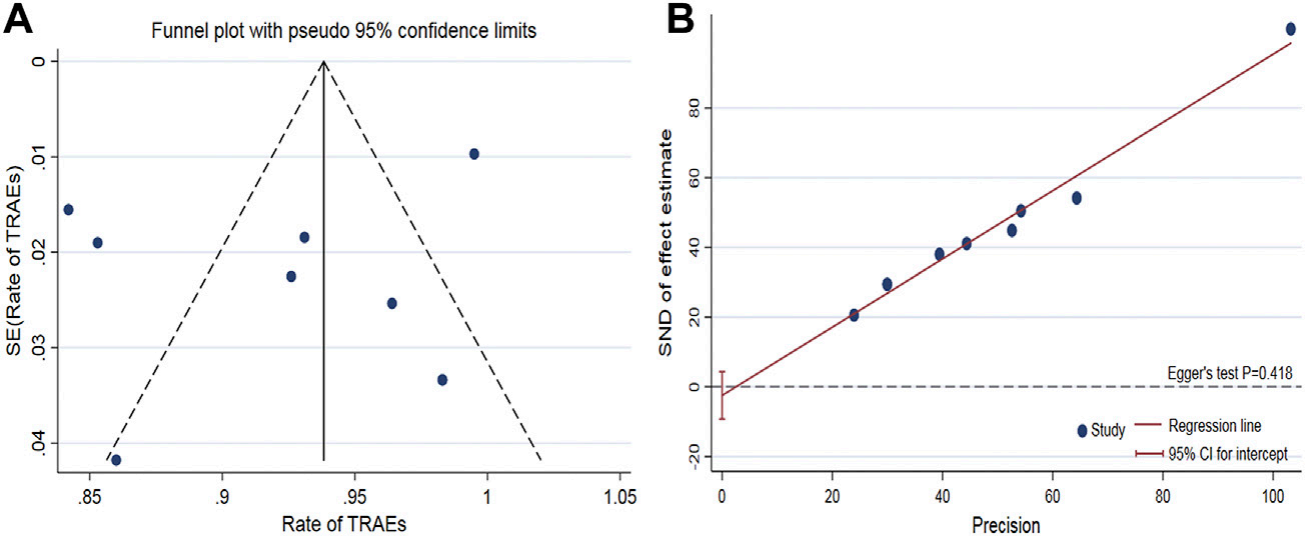
Funnel plot to assess publication bias of the pooled TRAEs **(A)**. Egger’s test to measure the funnel plot asymmetry of the pooled TRAEs **(B)**.

The funnel plot of the pooled percentage of improved PC and PD bother score did not show significant publication bias, and Egger’s test supports these results (Figures 4–6). Meanwhile, the funnel plot of pooled TRAEs revealed obvious publication bias (Figure 6A). However, given the significant heterogeneity of the pooled data on safety, Egger’s test is tough to interpret.

## Discussion

It was once thought that PD patients who are not surgical candidates should take oral monotherapy or observation for 6–12 months (Ziegelmann et al., 2020). Currently, although surgery has been regarded as the golden standard treatment for PD in the chronic phase, non-surgical therapy still appears to be an important option to reduce penile deformity and improve erectile function (Wymer et al., 2019). Meanwhile, various conservative treatments have been researched and are available for PD patients (Randhawa and Shukla, 2019). As the only FDA-approved drug, intralesional CCH is well explored and is more effective than oral or topical medication for PD (Gabrielson et al., 2018; Zhang et al., 2021). Meanwhile, CCH, officially approved in 2015 in Europe, was an important drug used for the intralesional therapy of PD by European experts in sexual medicine before (Porst et al., 2019). A preceding meta-analysis compared available intralesional therapies for PD and reported that intralesional CCH showed the best effect in terms of PC (Russo et al., 2019). However, this study did not compare all specific outcomes of treatment utilizing intralesional CCH, which may limit the assessment of results.

We performed a meta-analysis of prospective studies published in the post-FDA approval period in order to assess the efficacy and safety of intralesional CCH on PD in the stable phase. Based on current evidence, this meta-analysis supports the efficacy of intralesional CCH for the treatment of PD. We found an obvious improvement in PC and PD bother score from the pooled data, which is consistent with the results shown in previous clinical studies (Dhillon, 2015; Hoy, 2020). In particular, the pooled improvement in PC showed a remarkable advantage of intralesional CCH for PD. Meanwhile, due to the objectivity of measuring penile curvatures, the pooled information may support these results better in the evaluation of symptomatic improvement (Chen et al., 2018).

There are two major protocols involved in CCH therapy for PD: original treatment protocol and shortened protocol distinguished by different injection cycles and doses (Capoccia and Levine, 2018). Herein, although it is acceptable for some heterogeneity to exist in the pooled PD bother scores, we conducted subgroup analyses based on different protocols to determine the cause of heterogeneity. We found that dominant heterogeneity was observed in the subgroup with the original protocol. This might be attributed to the uneven distribution of the improvement of PD bother scores in the subgroup with the original protocol, which may be associated with the study design and unreported therapies prior to enrolling. Moreover, we also performed subgroup analyses stratified by a baseline penile curvature of 55°. It seems that PD patients with higher degrees of penile curvature would have more improvement in PD bother scores from intralesional CCH. All in all, the evidence from pooled PD bother scores appears to support the efficacy of intralesional CCH, let alone that this domain from the PD questionnaire is subjective. Despite the significant heterogeneity that existed in the pooled improvement of the PS score and PP score, it seems that intralesional CCH may work in amelioration of psychological and penile pain symptoms. However, considering that the penile curvature might be associated with psychological bother, we hold the opinion that the patients with PD may benefit more from the improvement of curvature deformity, including better mental status and decreased anxiety.

In this meta-analysis, the evaluation of clinical safety on intralesional CCH for PD revealed significant heterogeneity and obvious publication bias, even with sensitivity and subgroup analyses. While the majority of recipients with CCH therapy experienced mild TRAEs, previous clinical studies still demonstrated that intralesional injection of CCH was well tolerated (Dhillon, 2015). Yet, our analysis is not consistent with previous studies due to significant heterogeneity among included studies. We assume that this might be partially attributed to the discrepancy in safety evaluation, differences in baseline characteristics, and even differences in the follow-up that existed in the included studies. However, we also noted that most TRAEs were mild and could be settled without medical intervention. Meanwhile, serious TRAEs, including corporal ruptures and hematomas requiring surgical management, were seemingly rare as previously reported (Chung et al., 2020; Greear et al., 2020). Taken together, CCH injection is clinically safe for the treatment of PD. Moreover, due to intralesional injection of CCH is always used as part of a combination regimen for the treatment of PD, it is necessary to evaluate the clinical safety and drug interaction when combined with other agents.

There are some limitations to the present study. On the one hand, this meta-analysis was performed on the basis of published data from journal articles, which could inevitably introduce publication bias. Thus, we only included the RCTs and prospective clinical studies to decrease the risk of bias. On the other hand, in several included studies, some outcomes were unlikely to be extracted in a reasonable way or were simply not reported. Given the unavailable data in those studies, the endpoints of our study were assessed according to different included studies, which may affect the credibility and increase heterogeneity. Moreover, the best non-surgical treatment for PD is a combination model, including pharmacotherapy and mechanical therapy. Among included studies, CCH injection was combined with several mechanical therapies, such as vacuum therapy, penile traction, and modeling, which might have potential synergy in treatment (Li et al., 2018). Nevertheless, different models of combination therapy may have a negative influence on pooled efficacy. Additionally, we detected a significant heterogeneity and publication bias in the assessment of clinical safety. Therefore, the results of pooled data on safety should be interpreted with caution.

## Conclusion

In conclusion, the intralesional injection of CCH could significantly improve the penile curvature deformity of patients with PD. Meanwhile, CCH injection appears to ameliorate the PD bother score to some extent, and modified protocols may retain the same effectiveness. Moreover, despite the high incidence of mild TRAEs, the occurrence of serious TRAEs was rare, and overall safety seems to be acceptable. Future studies, especially RCTs are needed to clarify long-term effectiveness and the risk of complications. The comprehensive role of CCH injection combined with other agents requires further investigation through RCTs.
