# Supplementary figures and images for: The efficacy and safety of intralesional injection of collagenase Clostridium histolyticum for Peyronie’s disease: A meta-analysis of published prospective studies
Source: Front Pharmacol. 2022 Oct 5;13:973394. doi: 10.3389/fphar.2022.973394 (PMC9581257; doi:10.3389/fphar.2022.973394)

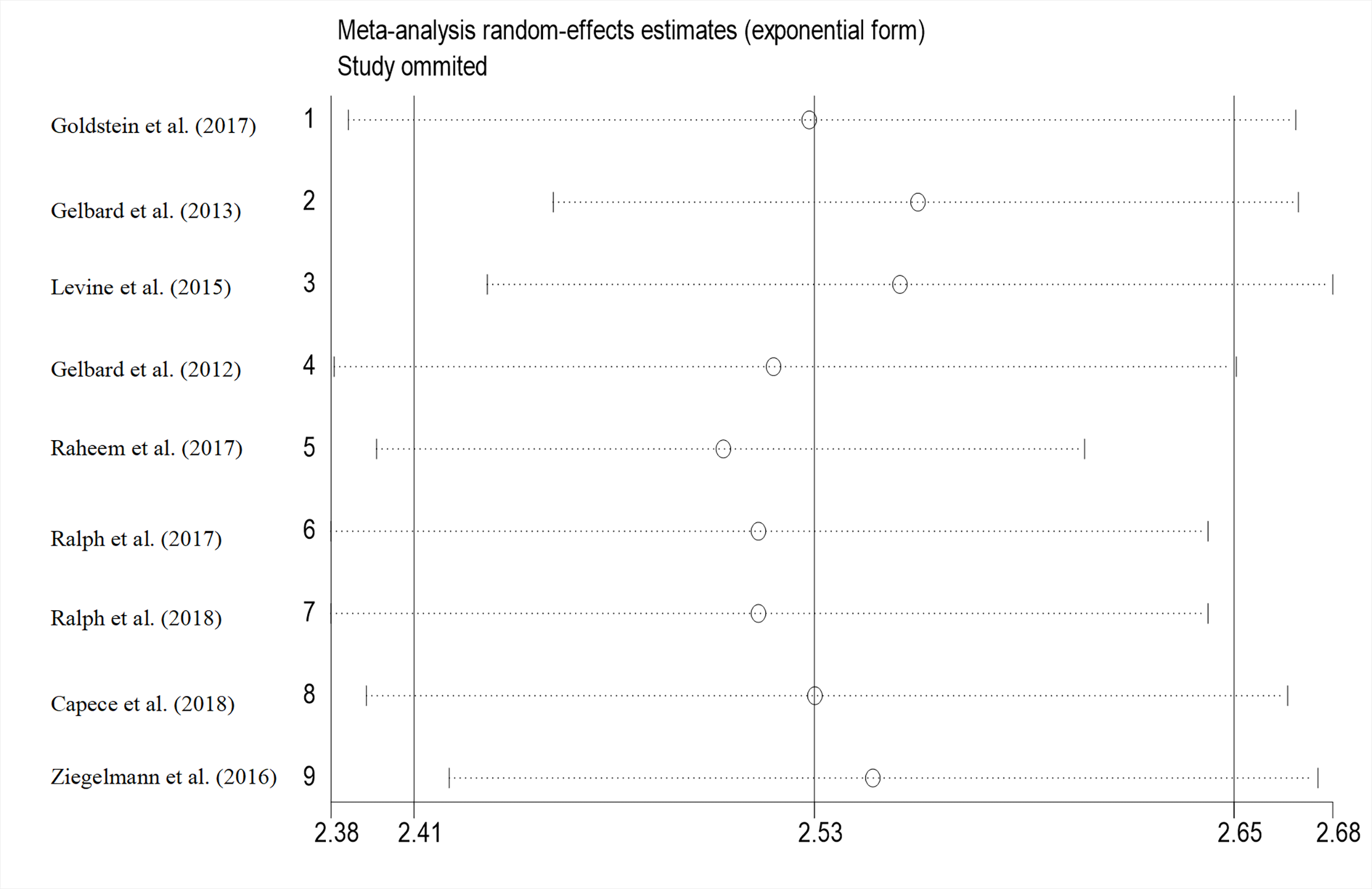

Supplement: Supplementary file 1 [file Image3.TIF]

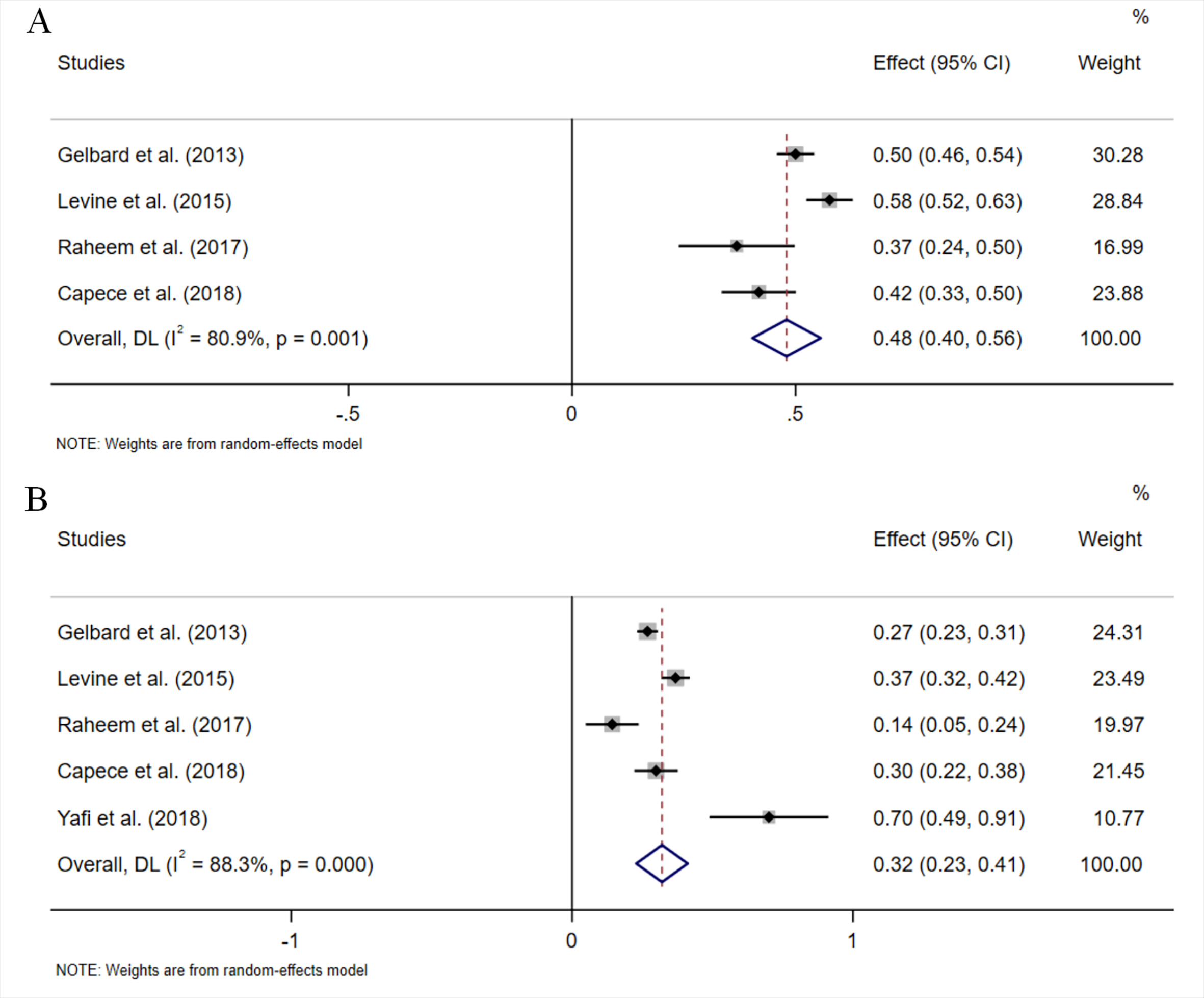

Supplement: Supplementary file 2 [file Image2.TIF]

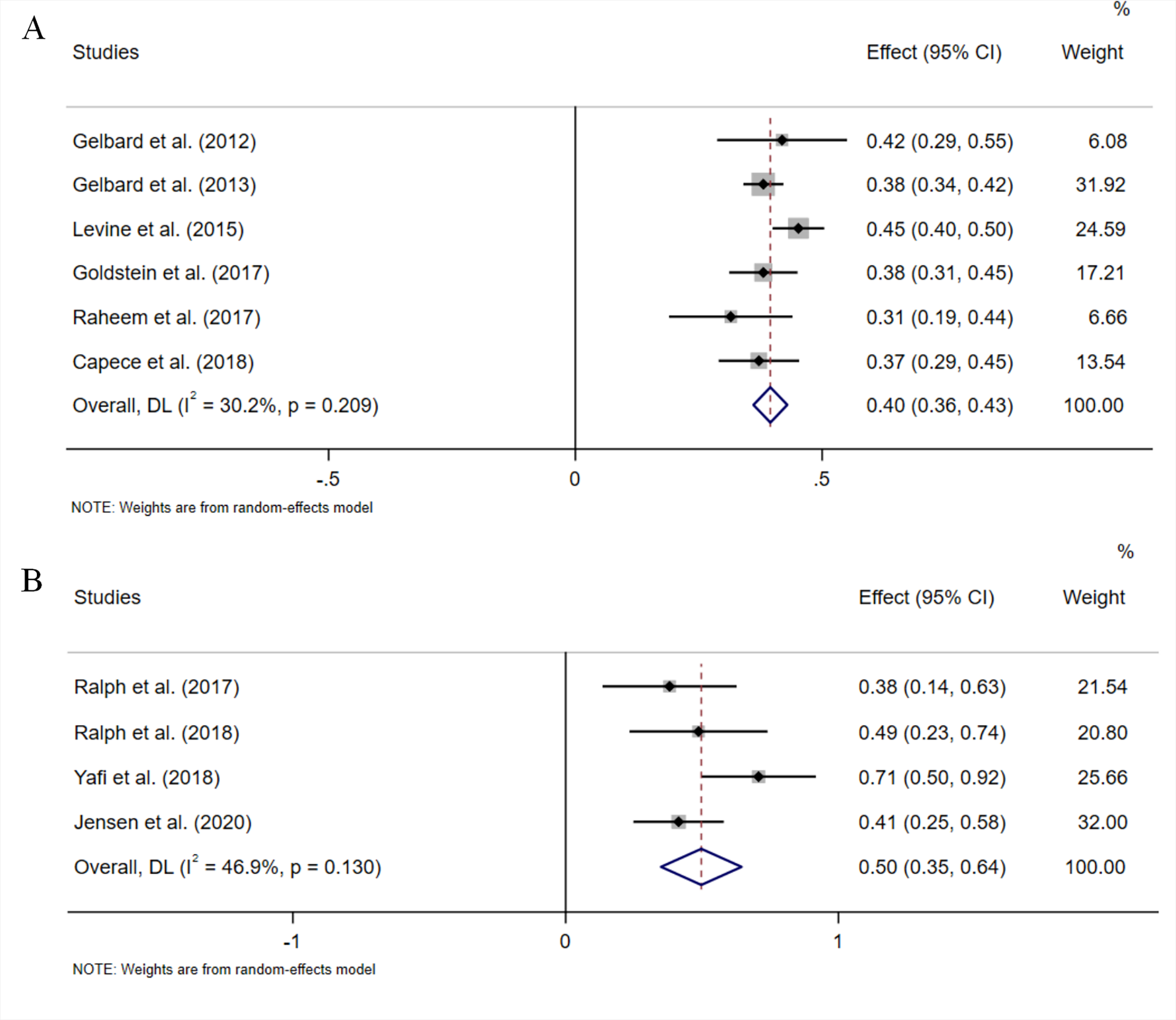

Supplement: Supplementary file 3 [file Image1.TIF]
